# Supplementary material for: Low coordination number copper catalysts for electrochemical CO2 methanation in a membrane electrode assembly
Source: Nat Commun. 2021 May 18;12:2932. doi: 10.1038/s41467-021-23065-4 (PMC8131708; doi:10.1038/s41467-021-23065-4)
Supplement: Supplementary file 1 — Supplementary Information [file 41467_2021_23065_MOESM1_ESM.pdf]

Supplementary Information

Low coordination number copper catalysts for electrochemical CO_2 methanation in a membrane electrode assembly

Yi Xu[1†], Fengwang Li[2†], Aoni Xu[2], Jonathan P. Edwards[1], Sung-Fu Hung[2,3], Christine M. Gabardo[1], Colin P. O'Brien[1], Shijie Liu[1], Xue Wang[2], Yuhang Li[2], Joshua Wicks[2], Rui Kai Miao[1], Yuan Liu[2], Jun Li[1, 2], Jianan Erick Huang[2], Jehad Abed[2,4], Yuhang Wang[2], Edward H. Sargent[2] & David Sinton[1]**

[1]*Department of Mechanical and Industrial Engineering, University of Toronto, 5 King's College Road, Toronto, Ontario, M5S 3G8, Canada.*

[2]*Department of Electrical and Computer Engineering, University of Toronto, 10 King's College Road, Toronto, Ontario, M5S 3G4, Canada.*

[3]*Department of Applied Chemistry, National Yang Ming Chiao Tung University, 1001 University Road, Hsinchu 30010, Taiwan.*

[4] *Department of Materials Science and Engineering, University of Toronto, 184 College Street, Toronto, Ontario M5S 3E4, Canada.*

[†]*These authors contributed equally.*

**Corresponding email: ted.sargent@utoronto.ca and sinton@mie.utoronto.ca*

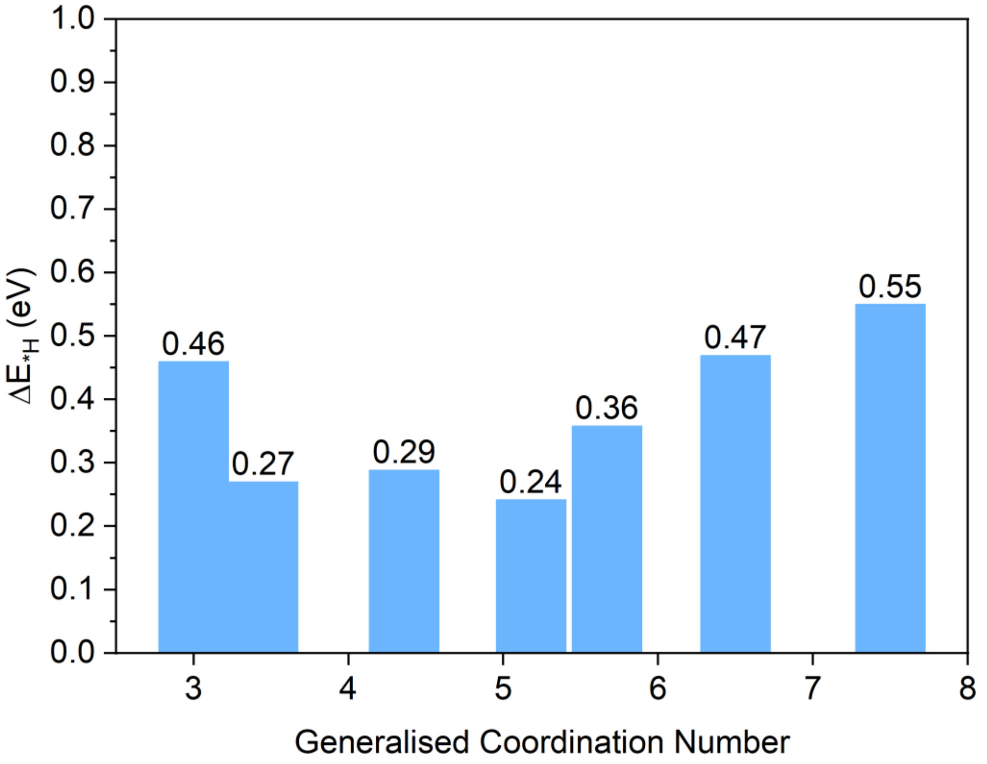

Supplementary Fig. 1 CO_2RR methanation DFT calculation. Reaction energies for *H adsorption on Cu catalysts of various generalised coordination numbers

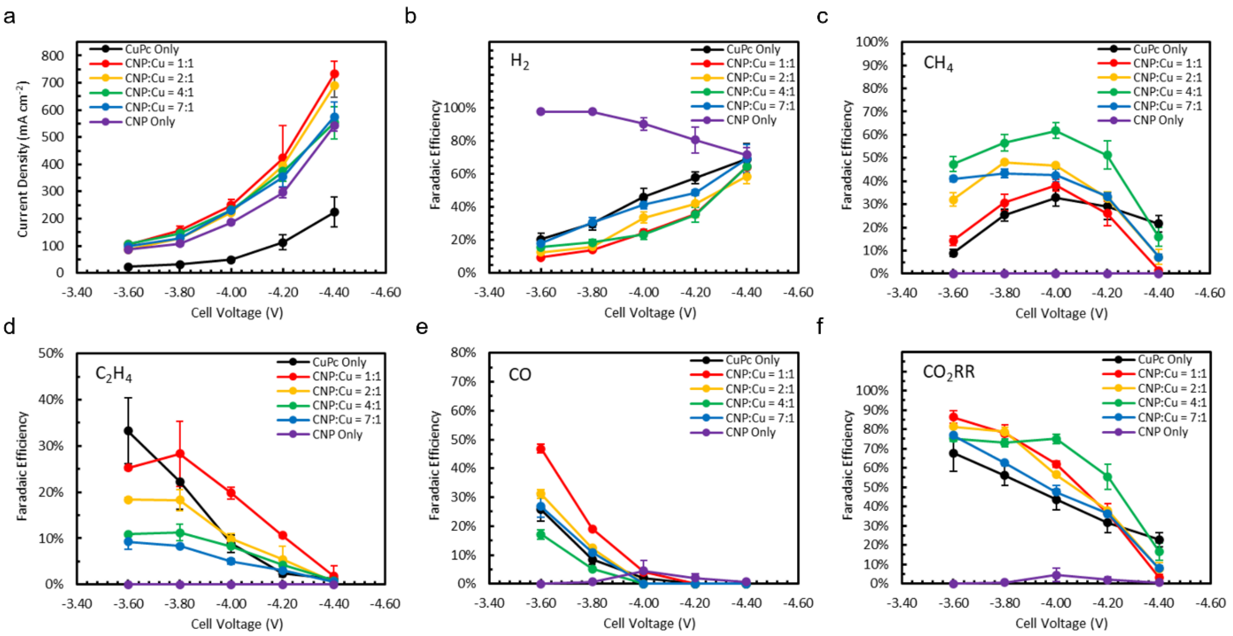

Supplementary Fig. 2 Effect of the CNP to CuPc ratio on CO_2RR methanation performance operating between full cell voltages of -3.6 and -4.4 V in an MEA system. a Current-voltage characteristics for samples with different CNP to CuPc ratios. **b** The Faradaic Efficiency (FE) toward hydrogen with different CNP to CuPc ratios. **c** The FE toward methane (CH_4) with different CNP to CuPc ratios. **d** The FE toward ethylene with different CNP to CuPc ratios. **e** The FE toward carbon monoxide with different CNP to CuPc ratios. **f** The FE of total CO_2RR products with different CNP to CuPc ratios. Error bars represent the standard deviation of three independent measurements.

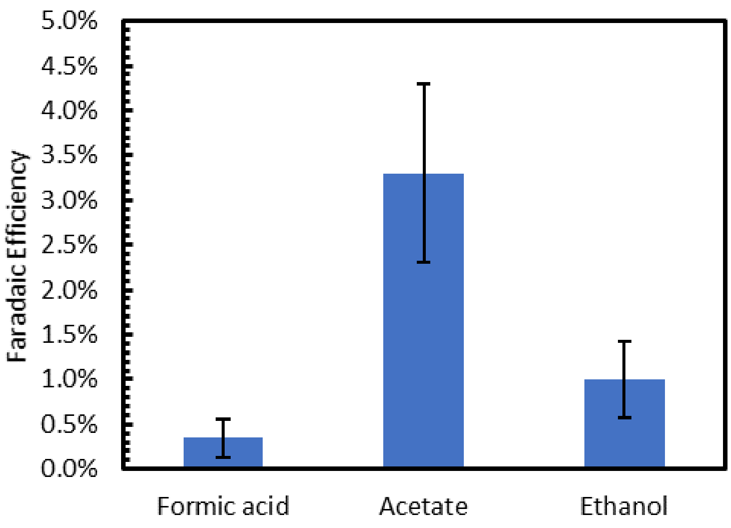

Supplementary Fig. 3 The FE toward liquid products with a 4:1 ratio of CNP to CuPc sample at -4.0 V in an MEA system. Error bars represent the standard deviation of three independent measurements.

Supplementary Note 1: In-situ X-ray absorption spectroscopy (XAS) Characterization

In-situ XAS was carried out under the same conditions as electrochemical testing using a modified flow cell with an opening in the gas chamber sealed by Kapton tape. The XAS signal was collected by the Vortex detector at the 9BM beamline of Advanced Photon Source (APS) in Argonne National Laboratory and the silicon drift detector at the 44A beamline of National Synchrotron Radiation Research Center (NSRRC). The scan range was kept in an energy range of 8800-9600 eV for the Cu K-edge measurements. The spectra were obtained by subtracting the baseline of the pre-edge and normalising that of the post-edge. K-edge extended X-ray absorption fine structure (EXAFS) analysis was conducted using Fourier transform on k^2-weighted EXAFS oscillations to evaluate the contribution of each bond pair to the Fourier transform peak.

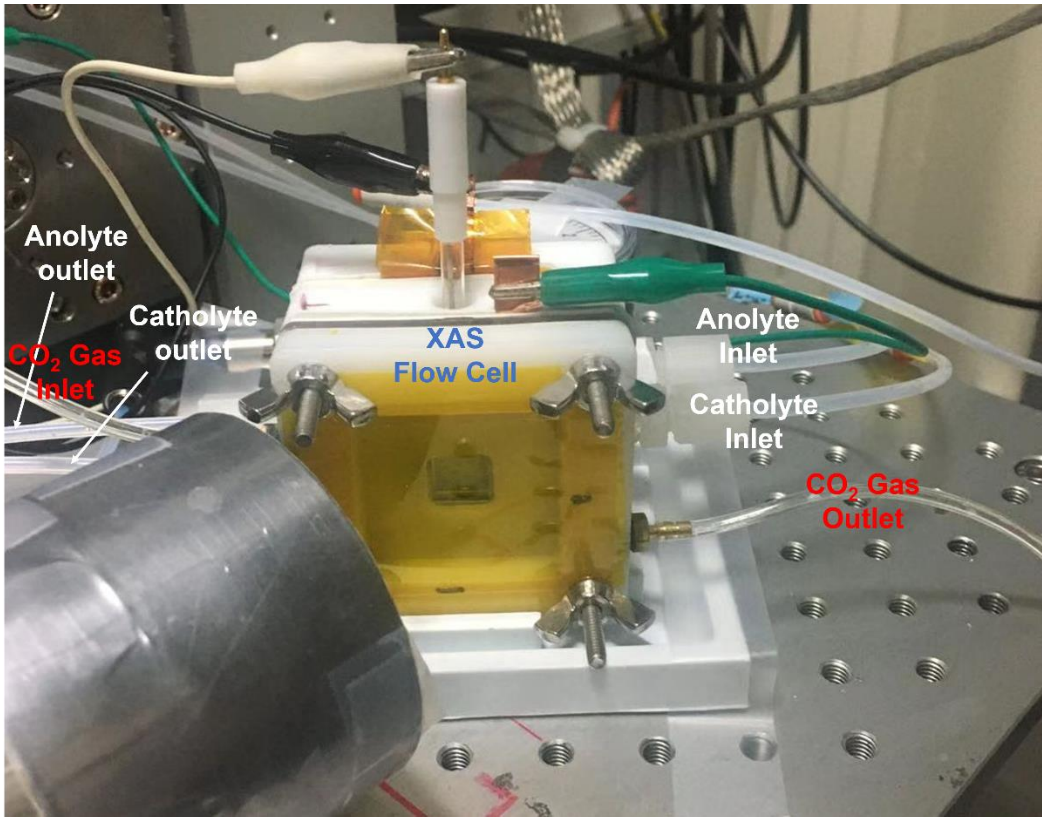

Supplementary Fig. 4 Photo of the in-situ XAS characterisation flow-cell electrocatalysis reactor.

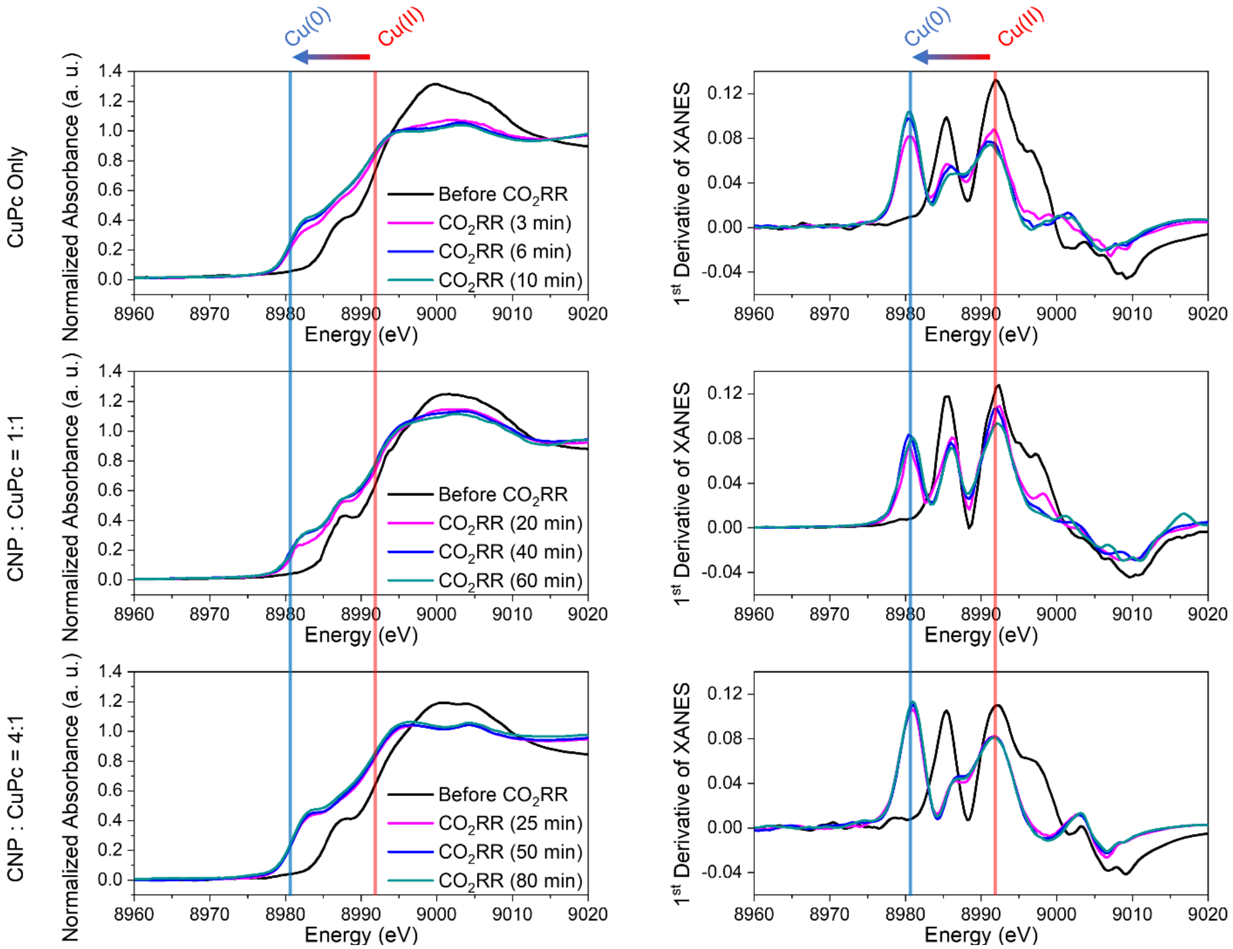

Supplementary Fig. 5 In-situ sample characterisation under electrocatalytic reaction conditions. Cu K-edge XANES spectra and first-order derivatives of the XANES spectra (collected at 200 mA cm^{-2} under CO_2RR conditions) for the sample containing **a** only CuPc **b** a 1:1 ratio of CNP to CuPc **c** a 4:1 ratio of CNP to CuPc.

Supplementary Table 1. The catalyst composition fitting from in-situ XANES sample characterisation. Sample compositions were determined by the linear combination fitting of X-ray absorption near edge structure (XANES) using copper foil and pure CuPc as the standards in the Athena software[1]. The fitting range is from -20 to 30 eV.

	CuPc Ratio	Cu Ratio
CuPC Only		
OCC	100.0%	0.0%
200mA (3 min)	26.8%	73.2%
200mA (6 min)	14.5%	85.5%
200mA (10 min)	9.8%	90.2%
CNP : CuPC = 1:1		
OCC	100.0%	0.0%
200mA (20 min)	62.7%	37.3%
200mA (40 min)	36.6%	63.4%
200mA (60 min)	12.8%	87.2%
CNP : CuPC = 4:1		
OCC	100.0%	0.0%
200mA (25 min)	19.2%	80.8%
200mA (50 min)	15.9%	84.1%
200mA (80 min)	12.3%	87.7%

REX2000 software using ab initio-calculated phases and amplitudes from the program FEFF 8.2 was used for the EXAFS fitting. The theoretical properties of the Cu-Cu and Cu-N paths, from the crystal information file of metallic Cu and CuPc, were incorporated to fit the experimental results. The ab initio phases and amplitudes were used in the EXAFS equation:

$$\chi(k) = S_0^2 \sum_j \frac{N_j}{kR_j^2} f_{eff_j}(\pi, k, R_j) e^{-2\sigma_j^2 k^2} e^{-\frac{2R_j}{\lambda_j(k)}} \sin(2kR_j + \phi_{ij}(k)) \qquad (1)$$

The neighbouring atoms with different distances were divided into j shells. N_j represented the coordination number of shell j at a distance of R_j relative to the central atom. $f_{effj}(\pi,k,R_j)$ was the ab initio amplitude function for shell j, while the Debye−Waller factor $e^{-2\sigma_j^2 k^2}$ accounted for the damping that resulted from static and thermal disorder in absorber−backscatterer distances. The mean free path term $e^{-\frac{2R_j}{\lambda_j(k)}}$ reflected losses due to inelastic scattering, where $\lambda_j(k)$ was the electron mean free path. The sinusoidal term $\sin(2kR_j + \phi_{ij}(k))$, where $\phi_{ij}(k)$ was the ab initio phase function for shell j, reflected the oscillations in the EXAFS. Shake-up/shake-off processes at the central atom(s) affected the amplitude reduction factor, s_0^2. *CN*, *R*, ΔE, and the EXAFS Debye−Waller factor (*DW*; σ^2) were variable parameters of the EXAFS equation for fitting the experimental result. The R range for fitting ranged from 1.20 to 2.80 and the k fell between 3.70 and 9.55.

Supplementary Table 2. Fitting parameters for the samples using metallic Cu-Cu and Cu-N paths as two-path fitting.

	Paths	*CN*	*R* (Å)	*ΔE* (eV)	*DW* ($Å^2$)	R-factor
Metallic Cu	Cu-Cu	11.3(2)	2.51(1)	0.8(4)	0.008(2)	0.011
	Cu-N	-	-	-	-	
CuPC	Cu-Cu	9.2(4)	2.54(3)	0.5(4)	0.009(2)	0.014
	Cu-N	0.1(0)	1.83(4)	-1.4(3)	0.007(1)	
C:Cu=1:1	Cu-Cu	6.9(2)	2.54(8)	-1.7(3)	0.009(0)	0.007
	Cu-N	0.2(1)	1.81(3)	3.8(3)	0.005(1)	
C:Cu=4:1	Cu-Cu	4.9(1)	2.53(6)	2.6(6)	0.008(1)	0.006
	Cu-N	1.2(2)	1.84(7)	-6.4(5)	0.006(4)	

Multi-path fitting was considered for Cu-Cu paths, Cu-N paths (within CuPc molecules), and Cu-C paths (interface between the metallic Cu cluster and CNP). The contribution from Cu-C paths was negligible and the fitting using two paths (Cu-Cu and Cu-N) matched well with the experimental spectra (**Supplementary Fig. 6**). This result confirmed that there were no detectable Cu-C paths formed once CNP was added. The fitting results were listed (shown in **Supplementary Table 2**) using only two-path fitting (Cu-Cu and Cu-N) to clarify these shells in the EXAFS spectra. These fitting results were also consistent with the XANES fitting using linear combination results.

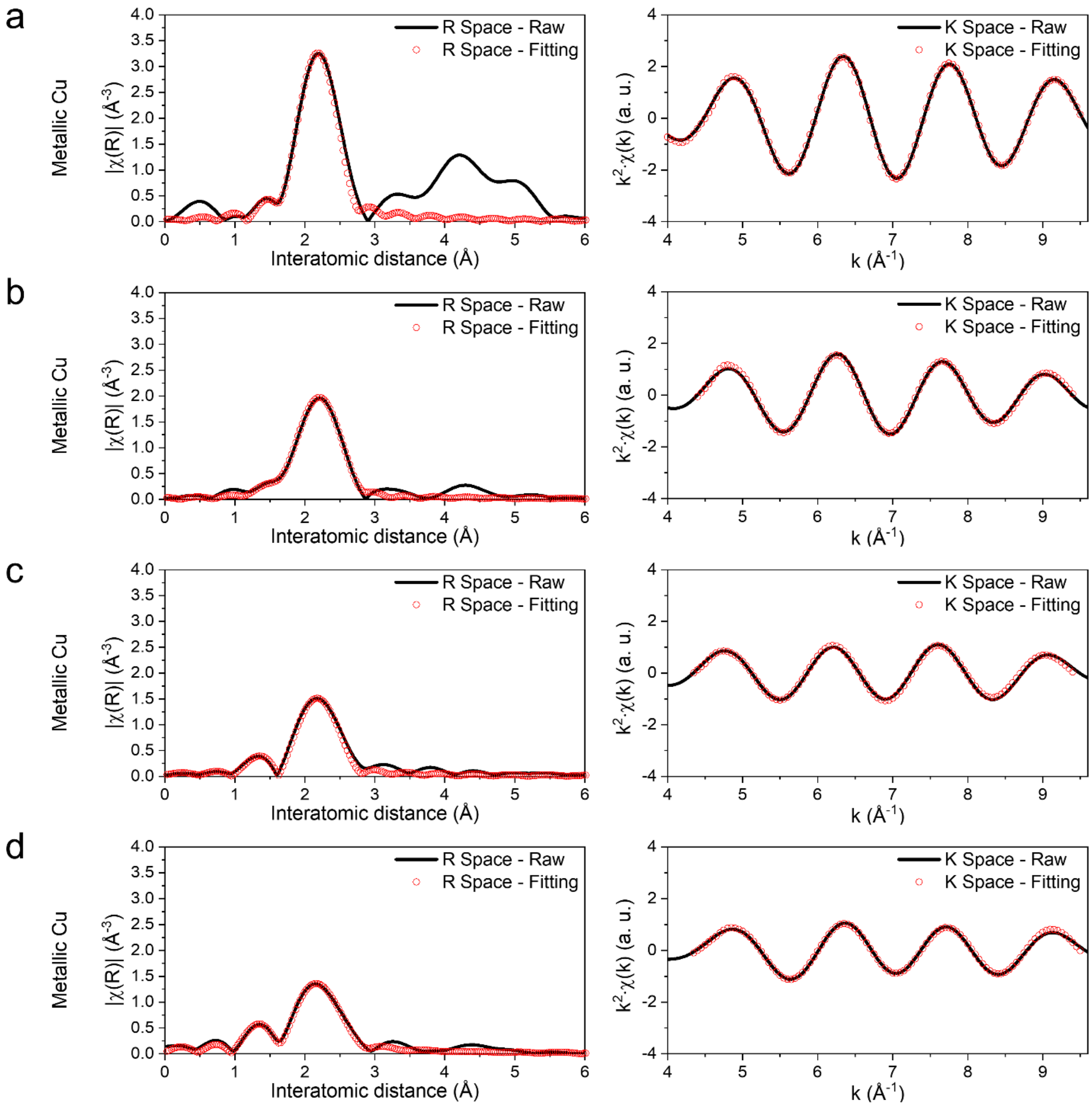

Supplementary Fig. 6 EXAFS spectra fitting results in R and k space. Cu EXAFS spectra R and k space fitting for the sample containing **a** a metallic Cu foil **b** only CuPc **c** a 1:1 ratio of CNP to CuPc **d** a 4:1 ratio of CNP to CuPc.

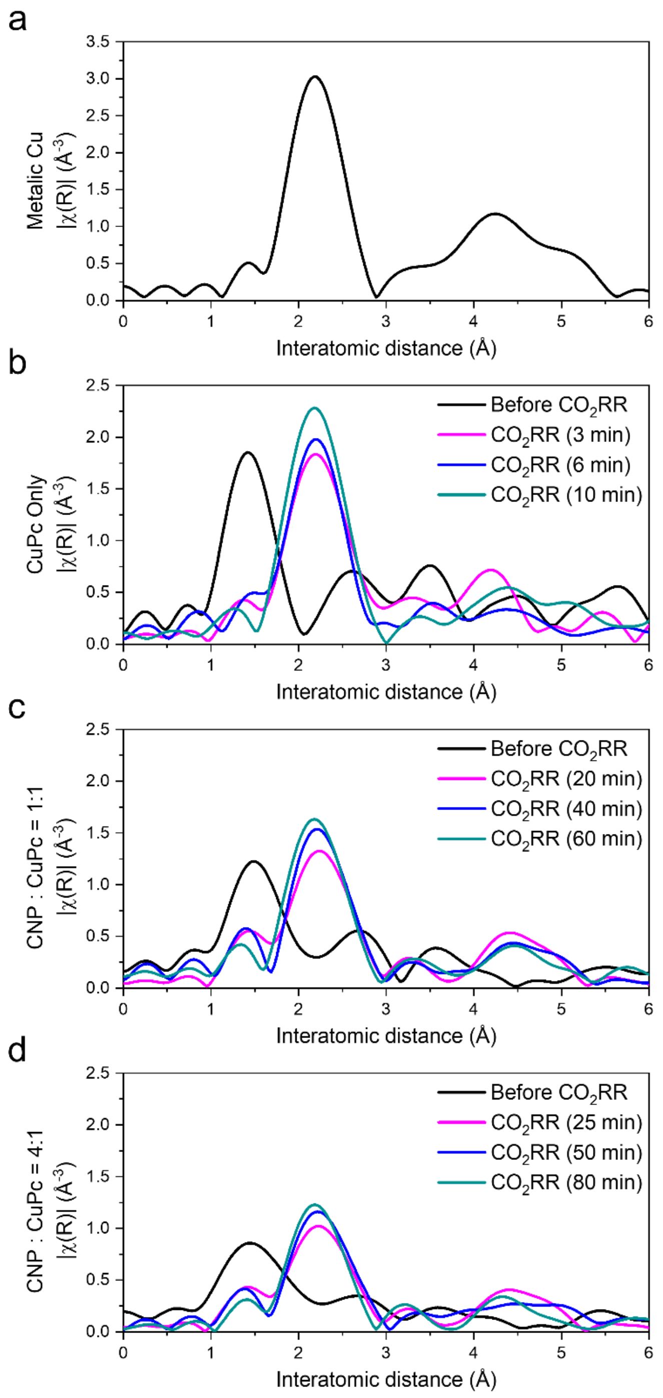

Supplementary Fig. 7 In-situ sample characterisation under electrocatalytic reaction conditions. Fourier-transformed Cu EXAFS spectra (collected at 200 mA cm^{-2} under CO_2RR conditions) for a sample containing **a** metallic Cu foil, **b** only CuPc, **c** a 1:1 ratio of CNP to CuPc **d** a 4:1 ratio of CNP to CuPc.

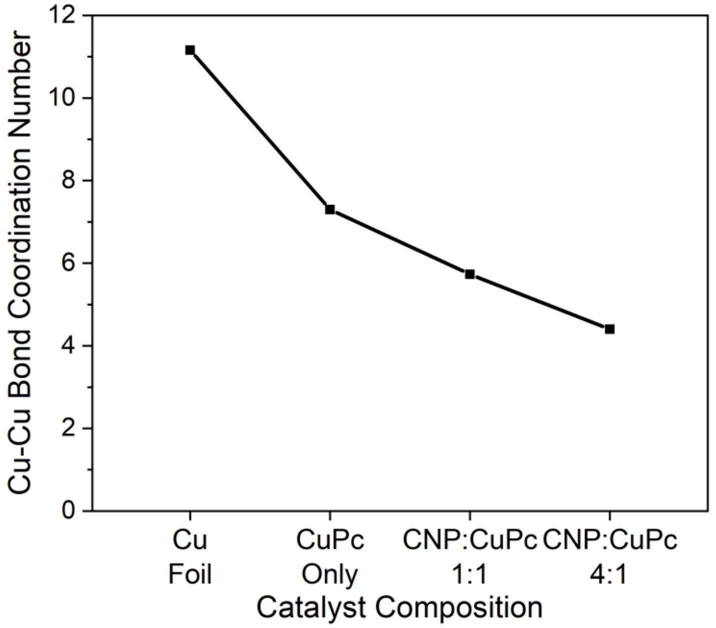

Supplementary Fig. 8 Comparison of the metallic Cu-Cu coordination number determined from EXAFS analysis for samples containing different ratios of CNP to CuPc and Cu foil sample.

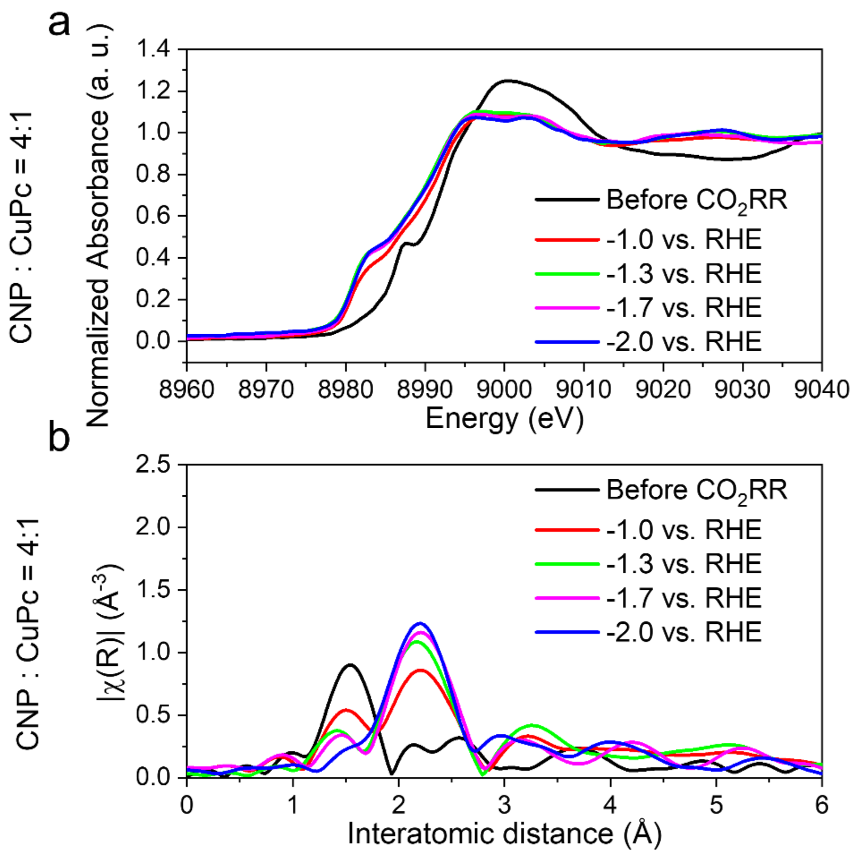

Supplementary Fig. 9 In-situ XAS characterisation under electrocatalytic reaction conditions, with voltage range from -1.0 V to -2.0 V vs. RHE (non-*i*R corrected), for a 4:1 ratio CNP to CuPc sample. a Cu XANES spectra**. b** Fourier-transformed Cu EXAFS spectra.

Ex-situ X-ray photoelectron spectroscopy (XPS) characterisation

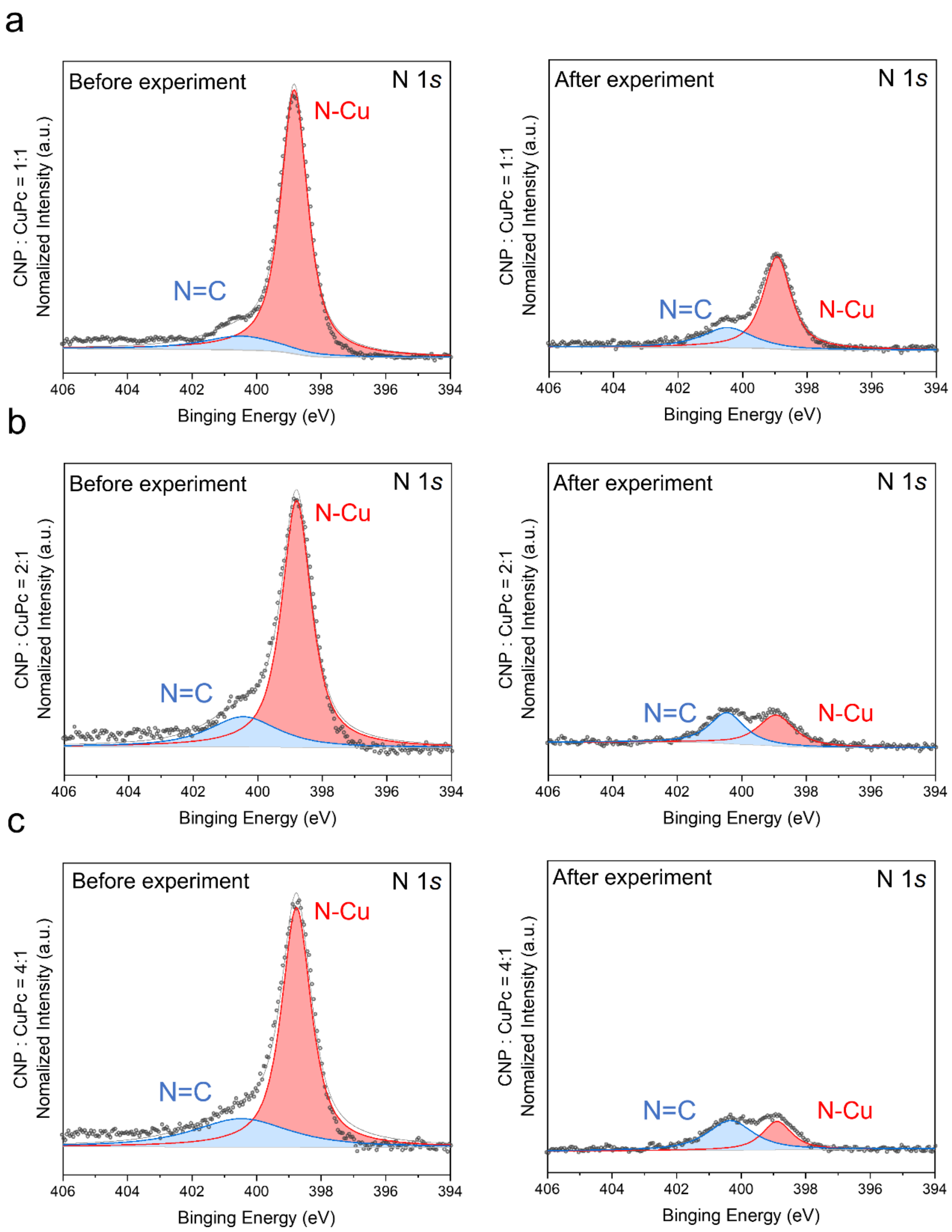

Supplementary Fig. 10 XPS characterisation of pre- and post-electrolysis samples for deconvolved N 1*s* peaks. Pre-electrolysis and post-electrolysis XPS spectra for a sample containing a **a** 1:1 ratio of CNP to CuPc **b** 2:1 ratio of CNP to CuPc **c** 4:1 ratio of CNP to CuPc.

Supplementary Table 3. XPS integrated areas for N 1s peaks. The N-Cu peak area normalised by the inert N=C peak area (the latter of which is assumed to not change during the reaction) was used to estimate the amount of CuPc remaining after the reaction.

XPS Sample	N=C Bond Absolute Peak Area	N-Cu Bond Absolute Peak Area	N-Cu Bond Normalised Peak Area	Percentage of N-Cu Bond Remaining
CNP:CuPc = 1:1 Before	5169.953	37777.295	7.307	36.3%
CNP:CuPc = 1:1 After	3308.329	8785.107	2.655	
CNP:CuPc = 2:1 Before	4842.720	18107.610	3.739	27.8%
CNP:CuPc = 2:1 After	4408.851	4583.436	1.039	
CNP:CuPc = 4:1 Before	6024.507	17582.035	2.918	21.7%
CNP:CuPc = 4:1 After	5472.815	3461.007	0.632	

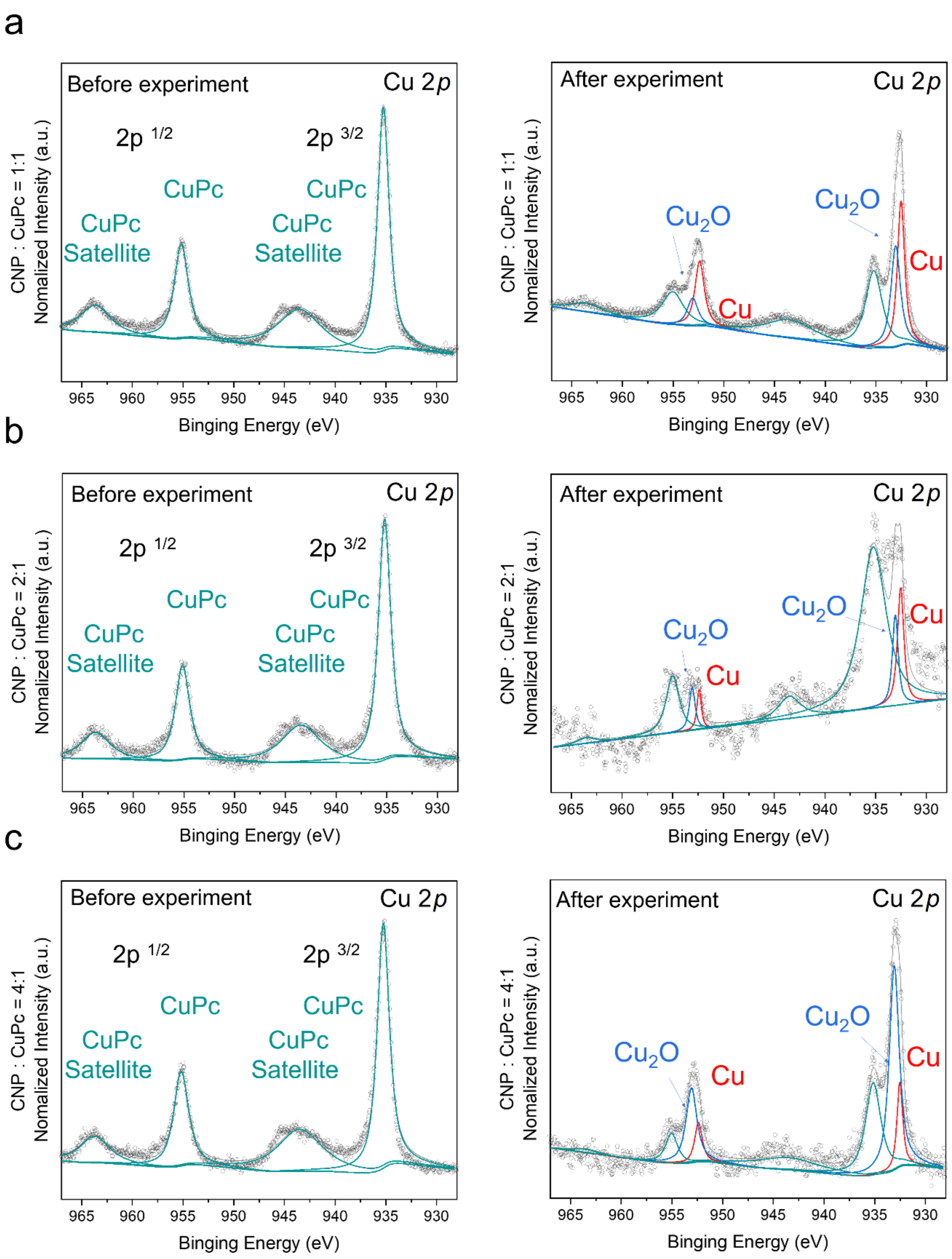

Supplementary Fig. 11 XPS characterisation of pre- and post-electrolysis samples for deconvolved Cu 2*p* peaks. Pre-electrolysis and post-electrolysis XPS spectra for a sample containing a **a** 1:1 ratio of CNP to CuPc **b** 2:1 ratio of CNP to CuPc **c** 4:1 ratio of CNP to CuPc.

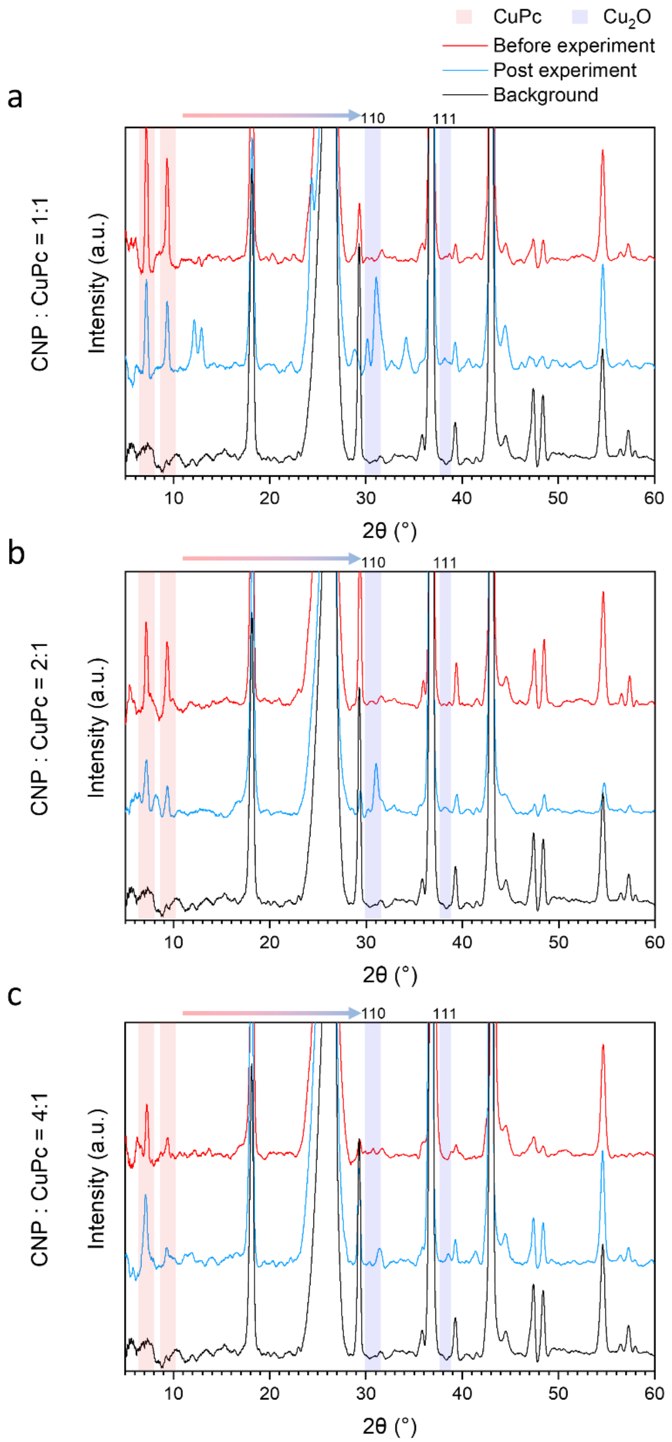

Supplementary Fig. 12 Ex-situ X-ray diffraction (XRD) characterisation of pre- and post-electrolysis samples. a XRD spectra for a sample containing a 1:1 ratio of CNP to CuPc, **b** a 2:1 ratio of CNP to CuPc, and **c** a 4:1 ratio of CNP to CuPc.

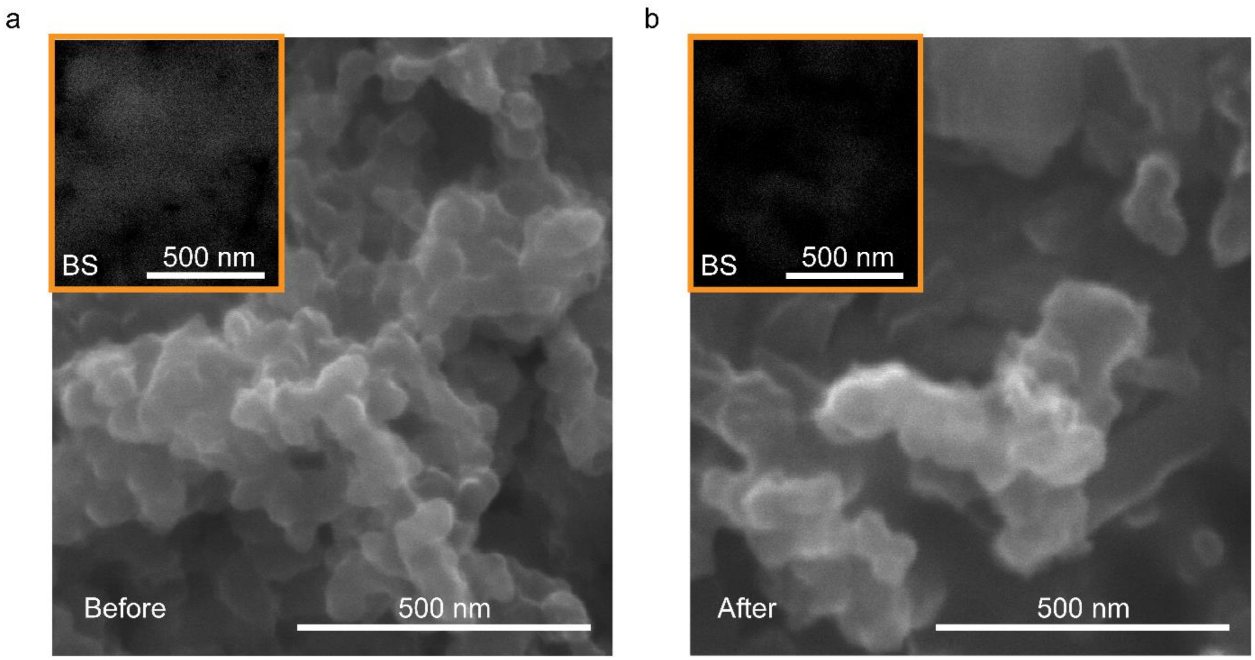

Supplementary Fig. 13 Scanning electron microscopy (SEM) and backscattered electrons (BS) images. All imaging is performed on 4:1 ratio of CNP to CuPc samples **a** Pre-electrolysis SEM image and BS image. **b** Post-electrolysis SEM image and BS image.

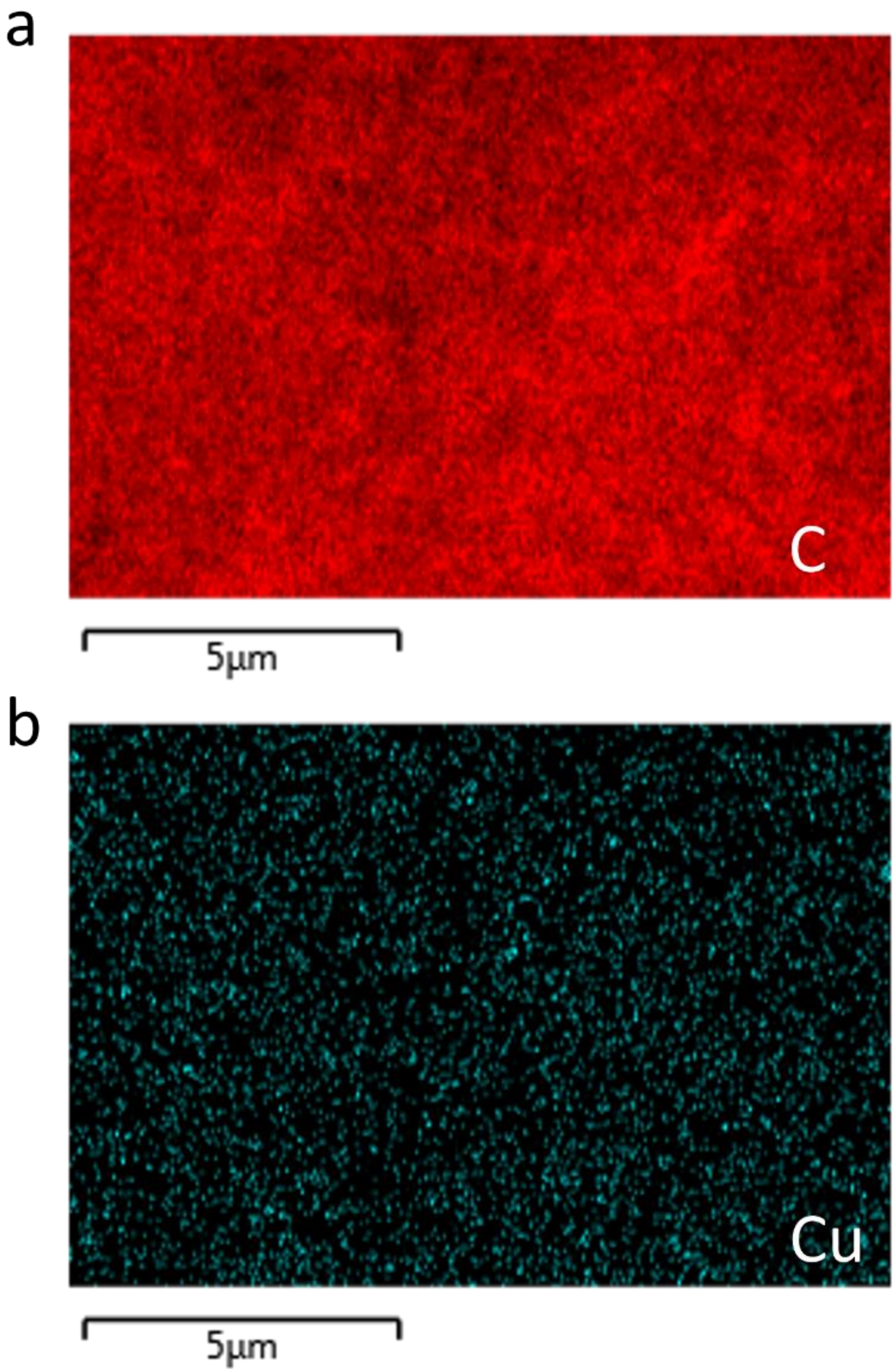

Supplementary Fig. 14 Scanning electron microscopy coupled with energy dispersive X-ray (STEM/EDX) spectroscopy images. All imaging is performed on a 4:1 ratio of CNP to CuPc post-electrolysis sample. **a** Carbon elemental mapping. **b** Cu elemental mapping.

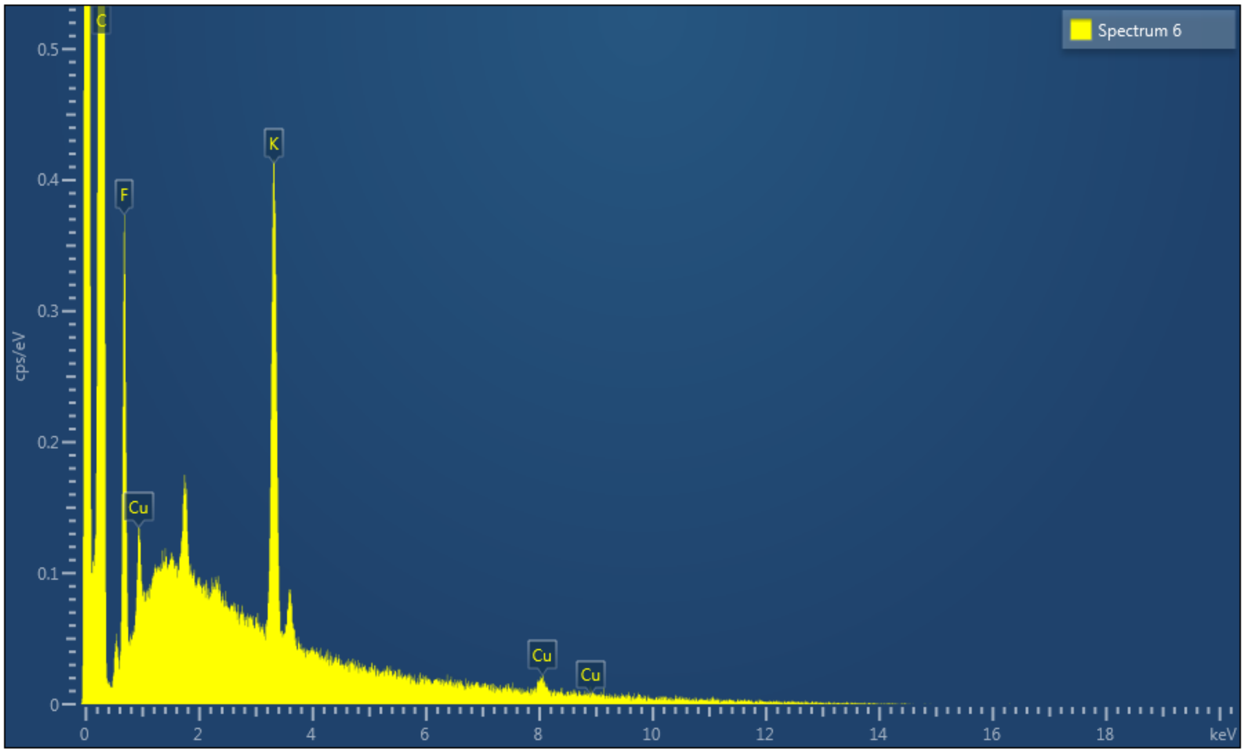

Supplementary Fig. 15 SEM/EDX spectra results performed on a 4:1 ratio of CNP to CuPc post-electrolysis sample.

Supplementary Table 4. SEM/EDX spectroscopy element analysis on a 4:1 ratio of CNP to CuPc post-electrolysis sample.

Element spectrum	Spectrum
C	96.73%
F	2.52%
K	0.63%
Cu	0.12%
Total	100.00%

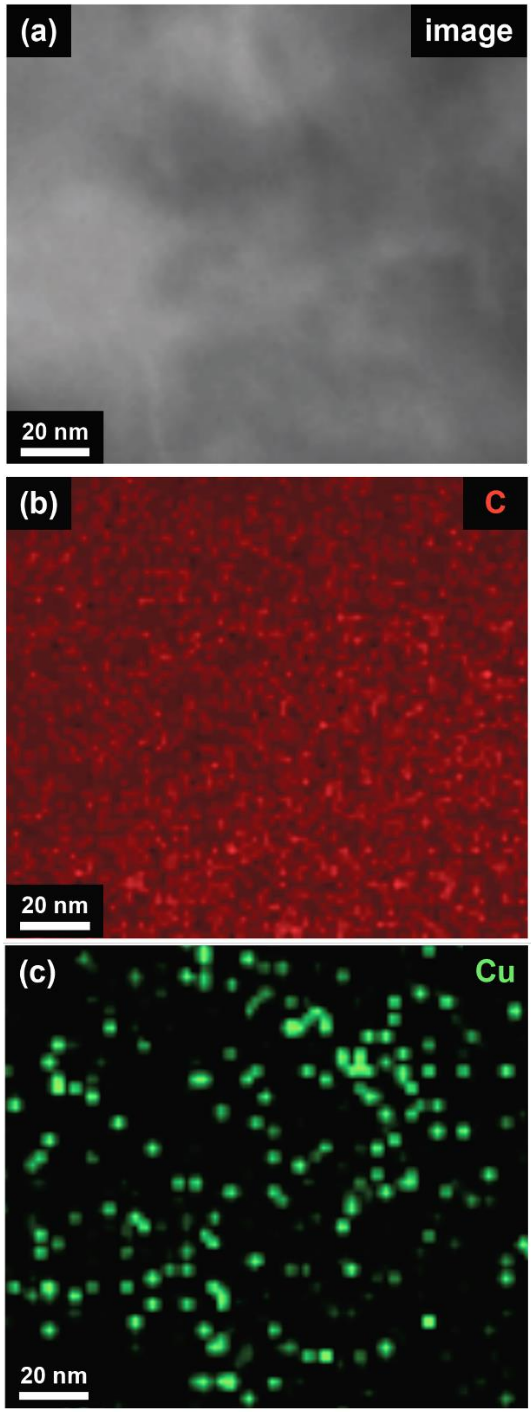

Supplementary Fig. 16 Scanning transmission electron microscopy coupled with energy dispersive X-ray (STEM/EDX) spectroscopy images. All imaging is performed on a 4:1 ratio of CNP to CuPc post-electrolysis sample. **a** STEM image. **b** Carbon elemental mapping. **c** Cu elemental mapping.

a

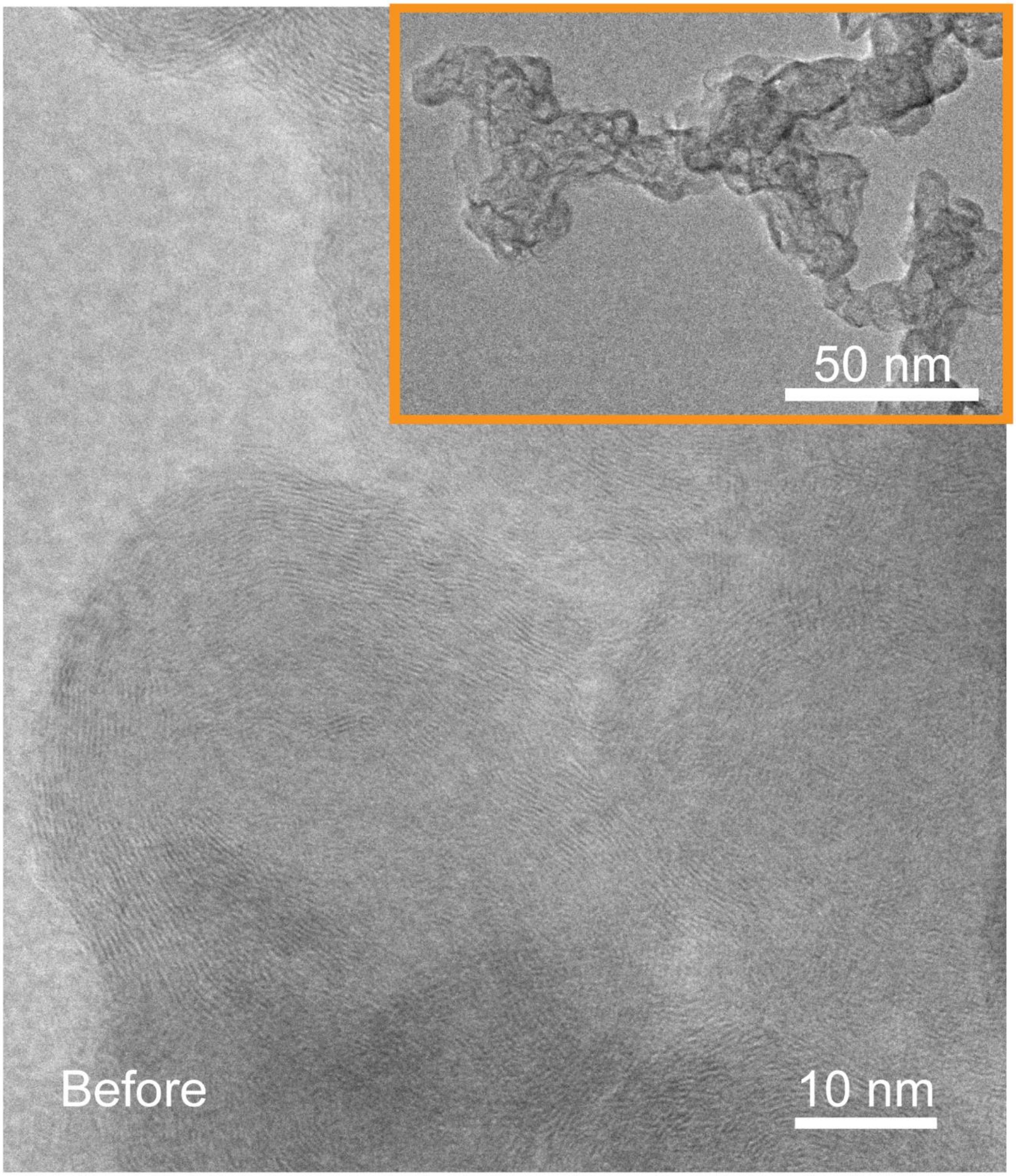

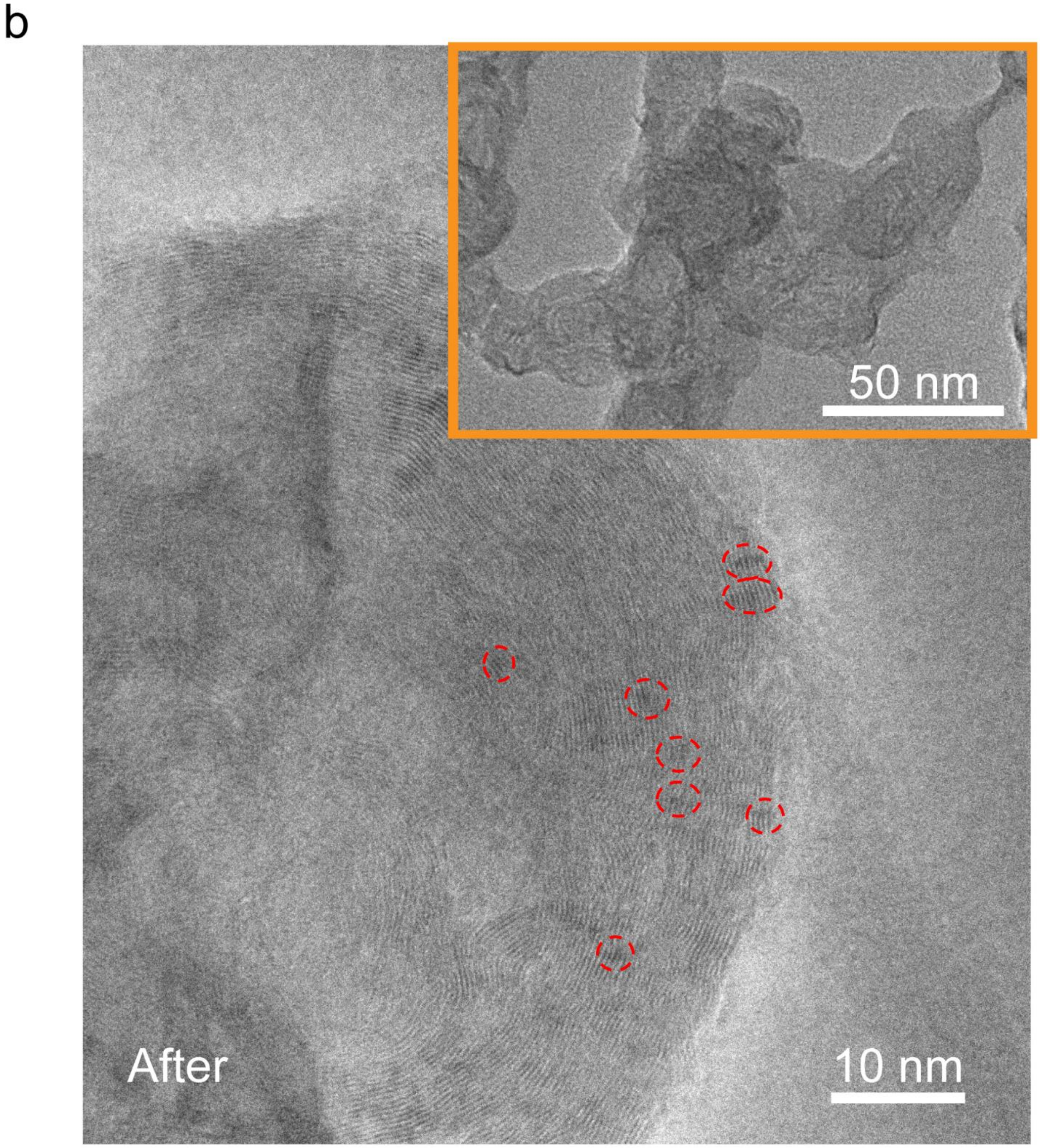

Supplementary Fig. 17 Transmission electron microscopy (TEM) images. All imaging is performed on 4:1 ratio of CNP to CuPc samples. **a** Pre-electrolysis TEM image. **b** Post-electrolysis TEM image.

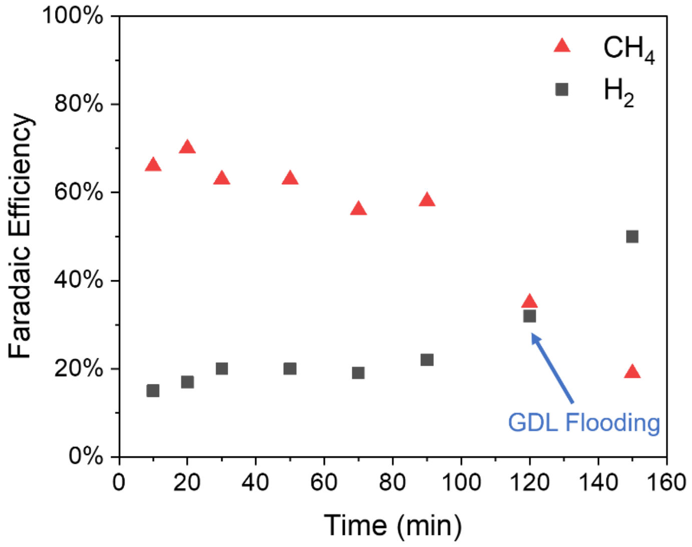

Supplementary Fig. 18 CO_2RR methanation performance operating at 200 mA cm^{-2} with 1M KOH electrolyte in a liquid flow cell configuration.

a

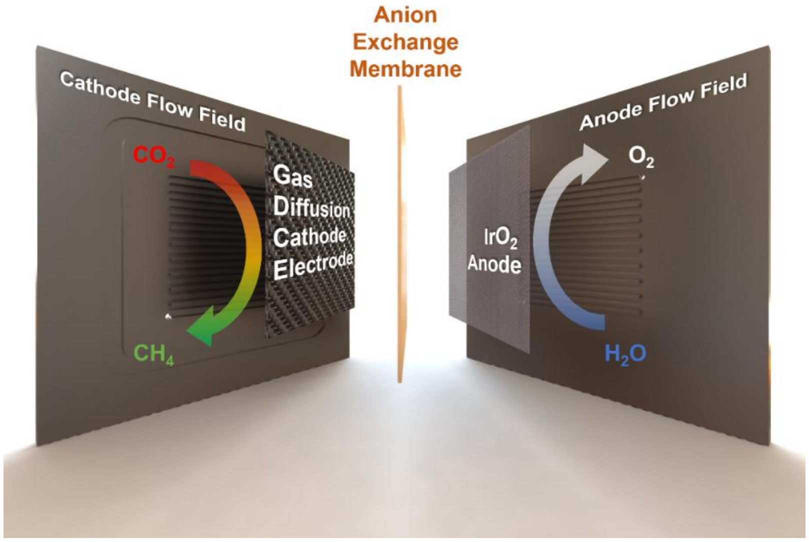

b

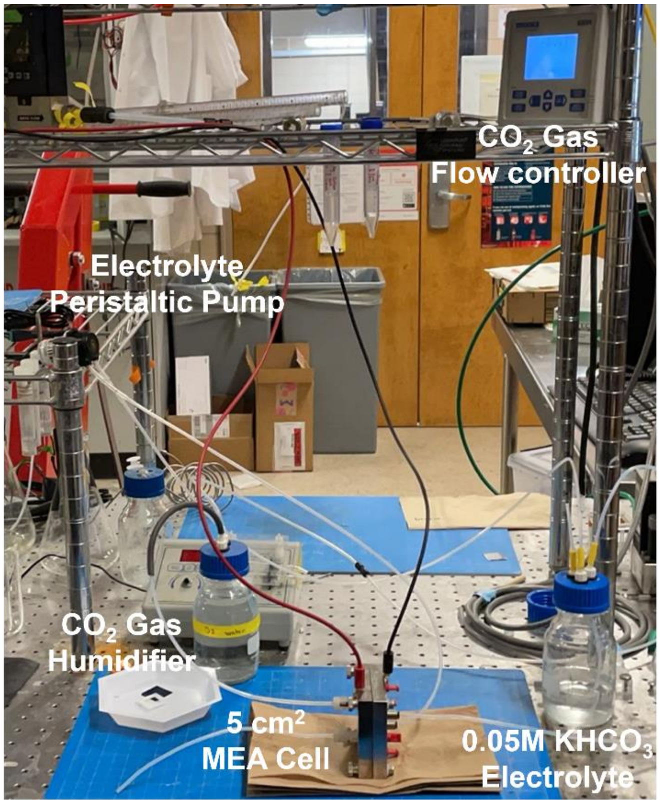

Supplementary Fig. 19 Membrane electrode assembly (MEA) CO_2 electrolyser. a Schematic of the MEA cell. **b** Photo of the MEA cell and associated system equipment.

Supplementary Table 5. The coordination number (CN) and generalised coordination number (GCN) of different configurations

Configurations	$\sum CN$	CN_{max}	GCN
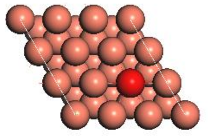	6*9+3*12	12	7.5
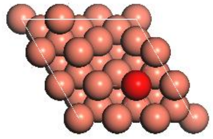	2*8+3*9+11+2*12	12	6.5
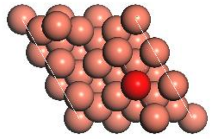	2*8+2*9+10+2*12	12	5.67
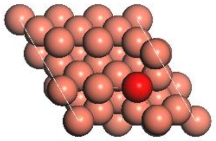	3*8+3*11	11	5.18
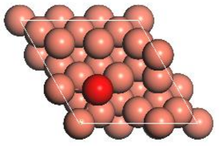	2*8+10+2*11	11	4.36
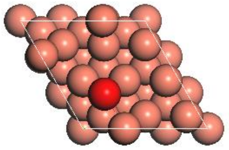	7+2*10+11	11	3.45
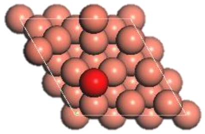	3*10	10	3

Supplementary Table 6. Vacancy formation energies of Cu surface models with various generalised coordination numbers

Generalised Coordination Numbers (GCN)	Configurations	Vacancy formation energy (eV)
7.5	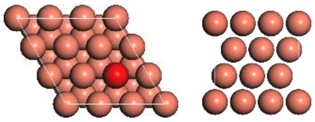	-
6.5	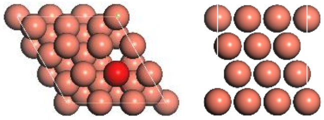	0.34
	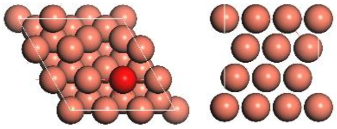	0.23
5.67	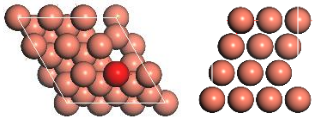	0.33
	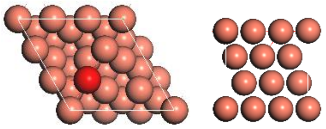	0.26
	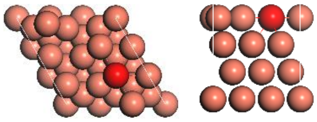	0.11
	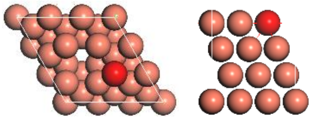	0.26
	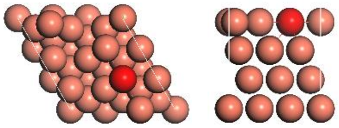	0.28
5.18	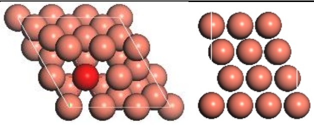	0.29
	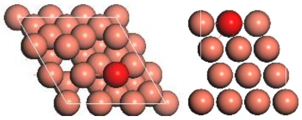	0.27

	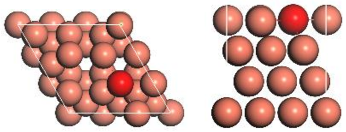	0.26
	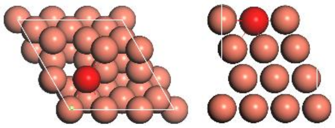	0.28
	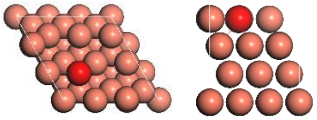	0.05
	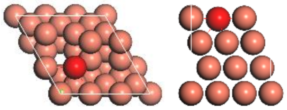	0.24
	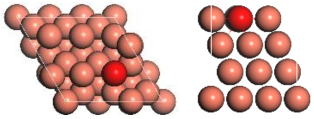	0.05
4.36	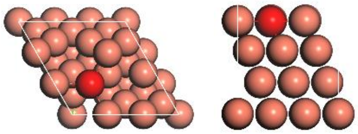	0.10
	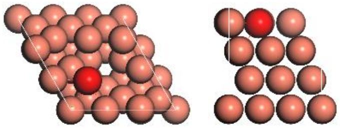	0.15
	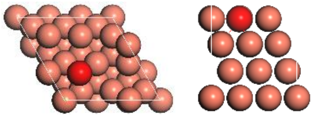	0.01
	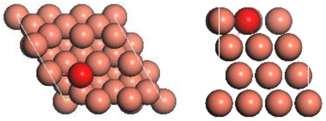	0.02
3.45	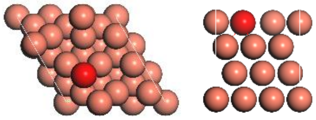	0.13
	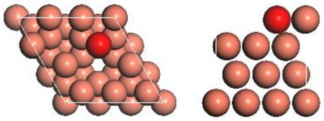	0.24
3	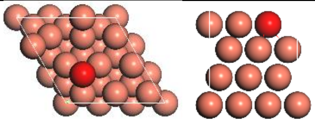	0.16

Supplementary Note 2: Vacancy formation energy calculation

The vacancy formation energies of various configurations, shown in **Supplementary Table 6**, were defined as,

$$E_v = \frac{1}{n}\left(E_{defect} - E_{no-defect} + n * E_{Cu}\right)$$

where E_v is the vacancy formation energy of modified Cu configuration, n is the number of Cu vacancies, E_{defect} and $E_{no-defect}$ represent the total energies of the defective and perfect model of the same component, and E_{Cu} is the energy of single Cu atom.

Supplementary Note 3: Reaction energy calculation

The hydrogenation of *CO on different catalysts surfaces was simulated according to the following reaction:

$$*CO + H^+ + e^- \rightarrow *CHO$$

where the * represents the adsorption site. Similarly, the C-C coupling process was simulated by:

$$*CO + *CO \rightarrow *OCCO$$

The adsorption of *H was calculated following,

$$H^+ + e^- \rightarrow *H$$

For each reaction, the free energies were given by the expression:

$$\Delta E = E_{prod} - E_{react}$$

where E_{prod} and E_{react} represent the total energies of reactant and product, respectively. Meanwhile, the energy of a proton was calculated using half of the total energy of H_2.

Reference

1 C. Da Silva-Cadoux, L. Zanella and J.-F. Gaillard, *J. Anal. At. Spectrom.*, 2012, **27**, 957.
